# Dynamic Computer-Assisted Surgery in Oral Surgery: A Systematic Review

**DOI:** 10.3390/jcm15020886

**Published:** 2026-01-21

**Authors:** Ariadna Requena-Gatell, Tania Moya-Martínez, Alba Sánchez-Torres, Eduard Valmaseda-Castellón, Rui Figueiredo, Esther Delgado-Molina

**Affiliations:** 1 Department of Dentistry, School of Medicine and Health Sciences, University of Barcelona, 08907 Barcelona, Spain; arequega85@alumnes.ub.edu (A.R.-G.); tmoyamar18@alumnes.ub.edu (T.M.-M.); albasancheztorres@ub.edu (A.S.-T.); eduardvalmaseda@ub.edu (E.V.-C.); ruibarbosa@ub.edu (R.F.); 2 Bellvitge Biomedical Research Institute (IDIBELL), Hospital Duran i Reynals, Gran Via de l’Hospitalet 199–203, L’Hospitalet de Llobregat, 08908 Barcelona, Spain

**Keywords:** dynamic computer-assisted surgery (dCAS), surgical navigation, mandibular third molar extraction, endodontic microsurgery, coronectomy

## Abstract

**Background/Objectives:** Dynamic computer-assisted surgery (dCAS) has emerged as a promising tool, particularly in implantology, enabling real-time procedural adjustments through 3D image-based tracking. However, their application in other areas of oral surgery remains limited. This systematic review aims to evaluate the advantages, limitations, clinical implications, and complications associated with the use of dCAS in oral surgery (excluding implants or miniscrew insertion) beyond implant placement, in comparison to conventional freehand (FH) techniques. **Methods:** A systematic review was conducted in accordance with the PRISMA guidelines. A focused PICO question was developed, and a comprehensive literature search was performed in PubMed, Scopus, and the Cochrane Library between February and March 2025, and supplemented by manual screening. The risk of bias of the included studies was evaluated using the Cochrane Risk of Bias tool (RoB 2) for randomized controlled trials (RCTs) and the ROBINS-I tool for non-randomized controlled trials (NRCTs). Data were summarized in tables and analyzed through qualitative synthesis. **Results:** Ten studies evaluating dCAS in several oral surgical procedures, including complex tooth extractions and endodontic surgery, were included. A substantial improvement was observed in accuracy of endodontic procedures. Operator experience was a key factor in surgical outcomes. Regarding postoperative complications, no significant differences were observed, although the trend indicated an equal or lower risk in comparison with conventional FH techniques. **Conclusions:** dCAS may significantly improve accuracy and efficiency in endodontic surgery and reduce operative time in complex mandibular third molar (M3M) extractions. The complication rate is comparable to that of conventional FH techniques. However, current evidence remains limited, heterogeneous, and mainly experimental. Further studies are recommended to validate the benefits of dCAS in clinical settings.

## 1. Introduction

In recent decades, oral and maxillofacial surgery has undergone notable advancements due to the progressive incorporation of digital technologies. In this context, the introduction of cone beam computed tomography (CBCT) has enabled the development of Computer-Assisted Surgery (CAS)—an innovative tool based on the use of three-dimensional (3D) imaging that allows guided surgical procedures to be performed according to virtual preoperative planning [1]. The first application of navigated surgery was reported in the field of neurosurgery in 1986 [2], with the aim of achieving more precise and less invasive surgery [3]. Over time, this technology has been applied to other health sciences, including dentistry and oral surgery.

CAS techniques are classified into two main categories: static computer-assisted surgery (sCAS) and dynamic computer-assisted surgery (dCAS). sCAS requires prefabricated surgical guides designed using 3D imaging and digital planning software [1]. These guides are adapted to the patient’s anatomy, allowing for more accurate instrument positioning and, consequently, higher precision [4]. Despite their advantages over freehand (FH) implant placement, sCAS present several limitations, including the inability to perform intraoperative adjustments, the requirement for space to accommodate the guides, sensitivity to guide displacement, longer surgery time, and higher costs, excluding the additional instrumentation needed for dCAS [5].

In this context, dCAS has emerged as an innovative tool that enables clinicians to virtually plan and guide surgery in real time by correlating 3D patient imaging with actual anatomical structures. This technology has the potential to enhance procedural precision and safety, offering a viable alternative to the limitations inherent in conventional techniques [4], particularly in complex procedures such as endodontic microsurgery and challenging surgical tooth extractions [6].

Despite these technological advances, most oral surgical procedures today are still carried out using conventional FH techniques. This conventional approach, largely dependent on the skill and experience of the operator, can compromise the consistency of outcomes, particularly in more complex cases [1].

Although dCAS has demonstrated high levels of accuracy and clinical reliability in dental implant placement [7,8,9] or miniscrew insertion [10,11], the available evidence regarding their application in other areas of oral surgery remains limited. For this reason, there is a justified need to carry out a systematic review of the use of dCAS beyond implant or miniscrew insertion, to determine their advantages, limitations, and implications for future clinical practice.

## 2. Materials and Methods

This systematic review was conducted in accordance with the PRISMA (Preferred Reporting Items for Systematic Reviews and Meta-Analyses) guidelines [12] and was registered in the international database of prospectively registered systematic reviews in health and social care (PROSPERO) under the number CRD420251151299. The following focused research question was formulated using the PICO framework: “Among patients undergoing oral surgical procedures, excluding implant or miniscrew placement, does dCAS enhance surgical accuracy, reduce procedure time, and minimize complications compared to conventional FH techniques?” (Table 1).

### 2.1. Eligibility Criteria

The inclusion criteria considered articles published within the last 10 years (from 2015 onward), including randomized controlled trials (RCTs) and non-randomized controlled trials (NRCTs) with postoperative follow-up, cohort studies, case series with more than 10 patients, as well as in vitro and cadaveric studies. Eligible studies were required to assess the accuracy and safety of dCAS in oral surgical procedures, not including implant or miniscrew placement.

Regarding exclusion criteria, cross-sectional studies, single case reports, and studies conducted on animal models were excluded. Additionally, no language restrictions were applied in the selection of articles.

### 2.2. Search Strategy

An electronic literature search was conducted by two independent reviewers (ARG and TMM) between 12 February and 18 March 2025, to identify studies meeting the predefined eligibility criteria (Table 2). Databases searched included PubMed (MEDLINE), Scopus (Elsevier), and the Cochrane Library (Wiley). Additionally, a manual search was conducted by reviewing the reference lists of the included studies to identify potentially relevant articles not retrieved through electronic databases.

### 2.3. Study Selection

Two independent reviewers (ARG and TMM) assessed the studies according to the predefined eligibility criteria. First, studies were selected by title and abstract. Then, the full text of eligible articles was read to finally assess their inclusion in the study. Disagreements were resolved through discussion with a third researcher (EDM). The Cohen’s kappa coefficient (κ) was used to assess agreement between the reviewers.

### 2.4. Risk of Bias Assessment

Version 5.1.0 of the Cochrane Handbook for Systematic Reviews of Interventions (RoB 2) [13] or the ROBINS-I tool [14] was used to assess the risk of bias in RCTs and NRCTs, respectively.

### 2.5. Data Extraction and Method of Analysis

Qualitative data from the selected studies was extracted and organized into tables summarizing the main characteristics of the included studies, the equipment, recording methods, and software used in each case, as well as outcomes related to surgical efficiency, accuracy, and complications observed in each study.

Surgical efficiency was defined as time-related parameters associated with the procedure, including preoperative planning time, navigation system setup time, operative time, and total procedure duration, when reported.

Surgical accuracy was defined as the deviation between the planned and the achieved surgical outcome, expressed through linear (2D and 3D deviations at the entry point and apex) and angular measurements, as reported in the included studies.

Studies were divided into:

- dCAS group: dynamic computer-assisted surgical navigation system that continuously tracks the real-time position of both the surgical instrument and the patient, using preoperative CBCT data for intraoperative guidance.
- FH group: Conventional method without the assistance of surgical guidance, such as static surgical guides or dynamic computer-assisted surgical navigation system. Accuracy outcomes rely on the clinician’s expertise and anatomical landmarks. All clinical cases in this group were assessed using CBCT imaging prior to the procedure, ensuring standardized preoperative planning.

## 3. Results

### 3.1. Study Selection

**Figure 1:**
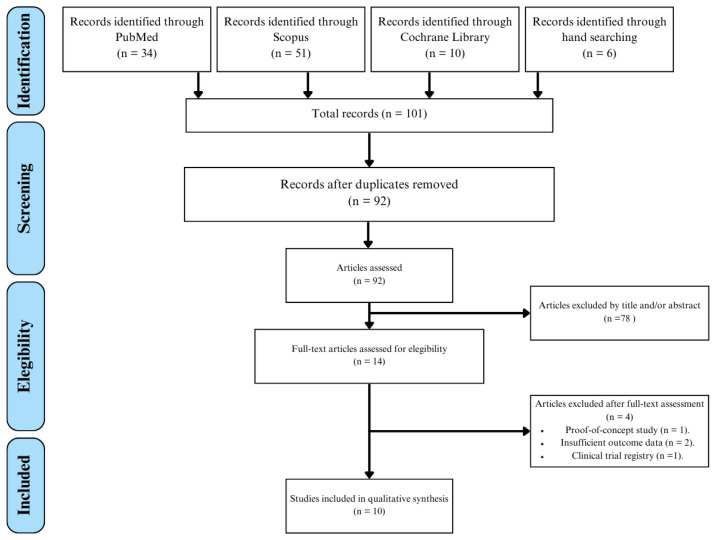
PRISMA flow diagram of the study selection process for the review.

A total of 101 articles were identified during the selection process, including 6 retrieved through manual search. Initially, 9 duplicate records were removed, followed by screening based on titles and abstracts. This process excluded 78 articles that did not meet the eligibility criteria. Full-text assessment of the remaining 14 studies led to the final inclusion of 10 articles in the qualitative analysis [15,16,17,18,19,20,21,22,23,24] including one article obtained through manual search [15] (Figure 1).

The selected studies comprised 2 RCTs, 1 NRCT, 1 observational study, 4 in vitro studies, and 2 randomized cadaveric studies. A Cohen’s kappa coefficient of κ = 1 was obtained.

### 3.2. Characteristics of the Included Studies

The results of the selected studies were extracted and organized into tables. Data such as study design, sample size, patient demographic characteristics, interventions performed, and assessed variables are presented in Table 3. Most clinical studies included individuals of both sexes and adults over 18 years of age, except for the study by Wang et al. [15], which included minors (age range: 7 to 28 years). The remaining 6 studies involved 3D in vitro models and cadaveric specimens.

The surgical interventions evaluated included complex mandibular third molar (M3M) extractions [16,17], coronectomies [18], supernumerary tooth (ST) extractions [15], as well as osteotomies and apical resections (RERs) [19,20,21,22,23,24]. The studies assessed outcome parameters such as surgical time, procedural accuracy, postoperative complications, and independent variables such as technique or operator experience.

The imaging system used in all studies to record anatomy and reference points was the CBCT. In each case, these data were integrated into the surgical planning software associated with each dCAS system, enabling precise 3D reconstruction of anatomical structures and virtual planning of surgical trajectories. Additional details on equipment, registration methods, and software used in the included studies are provided in Supplementary Table S1.

Regarding the registration system, fiducial marker-based registration was used to correlate the real patient (or model) with the virtual model, including custom-made splints or clips with radiopaque markers, among others. Markerless dCAS technologies were not applied in any case.

The dCAS systems employed include DHC-DI2^®^, IGI^®^, Dexter^®^, Brainlab ENT/CMF^®^, X-Guide^®^, DCARER^®^, and Navident^®^. The selection of equipment and associated software mainly depended on the type of surgical procedure performed as well as the experimental design.

### 3.3. Risk of Bias Assessment

The results of this assessment are presented in Supplementary Tables S2 and S3, which detail each individual study. Overall, the evaluation indicated a moderate to high risk of bias across the included studies. Most RCTs [15,17,19,23] exhibited unclear or high risk, primarily due to the lack of blinding of participants and outcome assessors, as well as inadequate allocation of concealment. In contrast, NRCTs [16,18] demonstrated serious risk of bias, mainly attributable to confounding variables and participant selection.

### 3.4. Qualitative Synthesis

#### 3.4.1. Efficiency

##### Tooth Extraction Procedures

All studies, except that of Emery et al. [16], assessed the efficiency of the dCAS based on the time required to complete the surgical procedure (Table 4). In the RCT by Xu et al. [17], the dCAS group required approximately 11 ± 1 min for preoperative planning and 4 ± 1 min for system setup. Although the surgical time was slightly shorter with dCAS than with FH, the overall duration of the procedure varied only minimally between the two groups, resulting in a similar operational benefit. In contrast, the studies by Zhang et al. [18] and Wang et al. [15] provided less detailed information, limiting the ability to evaluate variability and the actual impact of the system on procedure times.

##### Endodontic Surgery

In endodontic surgery, Aldahmash et al. [19], Martinho et al. [20,23], and Liu et al. [21] reported shorter osteotomy and RER times in the dCAS group compared with FH. It should be noted that all these studies were conducted using experimental models (cadaveric or in vitro), which may influence the generalizability of the findings. Tang and Jiang [22], as well as Martinho et al. [23], also explored the role of operator experience. In Tang and Jiang’s study, differences between the dCAS and FH groups were only evident among novice (NE) operators, suggesting a greater effect of the system in less experienced users. Conversely, Martinho et al. [23] observed similar procedure times within the dCAS group regardless of operator experience (EE vs. NE), indicating limited variability attributable to expertise when using this system.

Finally, in the study by Villa-Machado et al. [24], efficiency was assessed using specific indicators (visuomotor coordination time and drilling time), without reporting a global value for the total procedure duration. Consequently, it was not included in Table 4.

Overall, the efficiency outcomes summarized in Table 4 indicate that dCAS is associated with reduced operative time in specific procedural phases—most notably osteotomy and root-end resection—when compared with FH techniques. Although preoperative planning and system setup introduce additional time requirements, these were partially or fully offset by shorter intraoperative phases. Consequently, total procedure durations were comparable in clinical extraction studies, while experimental endodontic models demonstrated clear reductions in task-specific operative times.

#### 3.4.2. Accuracy

##### Tooth Extraction Procedures

Only Zhang et al. [18] evaluated the accuracy of dCAS in extraction procedures, specifically in M3M coronectomies. Accuracy was quantified by comparing planned versus actual surgical paths. The mean RMS deviation was 0.69 ± 0.21 mm, with maximum deviations of −1.87 ± 0.63 mm mesially and 1.45 ± 0.83 mm distally. Most deviations were small: 71.97% were <1 mm, 22.96% fell between 1–2 mm, and only 4.52% reached 2–3 mm. These values indicate consistently low spatial error and support the high procedural precision of dCAS in M3M coronectomy.

##### Endodontic Surgery

Table 5 summarizes the accuracy of outcomes reported in endodontic surgery studies. Across all included studies, accuracy was evaluated using linear and angular deviation metrics, although the specific parameters varied between studies. Overall, dCAS consistently demonstrated smaller 2D and 3D deviations than FH, reflecting meaningful reductions in spatial error rather than differences limited to statistical thresholds. Angular deflection values followed the same pattern, with lower angular deviations in the dCAS groups across all studies, indicating improved control over instrument trajectory.

Several studies further stratified outcomes by operator experience. In these analyses, expert operators generally achieved smaller deviations and narrower variability ranges than novice operators. Importantly, the magnitude of the difference between expert and novice performance tended to be larger under FH, whereas dCAS reduced the spread of operator-dependent variability, suggesting that dynamic navigation may help standardize accuracy across different experience levels.

#### 3.4.3. Complications

Reporting complications across the included studies was heterogeneous. Even though most studies recorded the total number of mishaps occurring during the procedure, it was not always explicitly stated whether the reported events corresponded to individual patients or individual procedures.

##### Tooth Extraction Procedures

Among tooth extraction procedures, complications were reported exclusively in clinical studies (Table 6). Three of the ten studies documented no adverse events in the dCAS group. Emery et al. [16] included only cases with a complexity score ≥ 9 according to the Juodzbalys and Daugela classification system (0–18 scale) [25], thereby excluding less-complex extractions. A total of 12 complications were recorded among the 25 extractions performed with dCAS, with postoperative infection being the most frequent (*n* = 7). Among the studies included in the table, the reporting of complications as per patient or per procedure is specified in all cases; however, one study does not report complications, and two studies do not clearly indicate whether complications are reported per patient or per procedure.

##### Endodontic Surgery

In the context of endodontic surgery, all reported complication data refer to per-procedure outcomes and were obtained from experimental studies. Only Villa-Machado et al. [24] identified differences between subgroups when assessing the influence of operator experience with the navigated system (dCAS EE = 18 vs. dCAS NE = 32). The most frequent complication was incomplete RER (26 cases; 21.7%), followed by excessive resection (11 cases; 9.2%) and overpenetration (11 cases; 9.2%).

## 4. Discussion

This systematic review evaluates the current scientific evidence regarding the accuracy, effectiveness, and current limitations of dCAS in oral surgery, beyond their application in the field of implantology and miniscrew insertion, where multiple studies have already demonstrated their benefits [7,8,9]. The included studies are highly heterogeneous, comprising RCTs, non-randomized clinical studies, cadaveric studies, and in vitro experiments.

Accuracy in oral surgery is a critical factor of therapeutic success, contributing to the reduction of surgical risks and both intraoperative and postoperative complications. Despite ongoing technological advances, procedures such as complex extractions or endodontic microsurgery remain challenging due to limited accessibility, restricted visibility, and the proximity of critical anatomical structures. In this context, dCAS offers a promising alternative to conventional FH techniques, providing enhanced spatial control, reduced dependence on operator experience, and greater intraoperative adaptability [6].

A key step in navigated surgery is the accurate registration of the patient’s anatomy, which can be achieved using either fiducial marker-based and markerless techniques. In the field of implantology, markerless approaches have gained prominence over marker-based methods [26]. These rely on anatomical landmarks, such as bony prominences or dental cusps, eliminating the need to fabricate a splint or clip, thereby enhancing patient comfort. However, outside the context of implant placement, clinical studies validating the accuracy outcomes of both techniques are still lacking. Notably, all studies included in this review employed fiducial markers for anatomical registration, meaning that the reported accuracy outcomes cannot be directly extrapolated to markerless navigation systems. Jorba et al. [27] evaluated the impact of different registration methods using dCAS on implant placement accuracy and reported higher accuracy compared with marker-based registration, without an increase in surgical time. As these emerging tools may influence accuracy outcomes, further investigation is needed to validate their effectiveness in other fields of oral surgery beyond implantology.

On the other hand, an additional factor to consider is the efficiency of the technique employed. Several studies in implantology have reported that the use of dCAS can extend the duration of the procedure by approximately 14 min compared to the conventional FH technique, and in some cases, even double the usual operative time [28,29]. However, this trend does not appear to be consistent in other areas of oral surgery. In the context of complex dental extractions, Xu et al. [17] reported that, for deeply impacted horizontal M3Ms, the surgical time in the dCAS group was 22 ± 3 min compared with 36 ± 3 min in the FH group, representing a 14 min reduction. Although dCAS requires additional time for preoperative planning and system setup, the overall procedure duration remained comparable between groups. This indicates that dCAS can streamline specific intraoperative phases while preserving overall procedural efficiency. Shorter surgical times may offer logistical and economic advantages for clinicians, enhance patient satisfaction and postoperative recovery, and reduce the risk of complications.

In this regard, in vitro and cadaveric studies in endodontic surgery have also shown a reduction in surgical time when using dCAS. However, given the experimental nature of these designs, these findings reflect technical feasibility rather than confirmed clinical efficiency.

In procedural phases such as osteotomy or RER, which are considered the most time-consuming, time reductions of up to 50% have been reported in certain cases [19,20,21,23], aligning with outcomes from previous cadaveric studies [30]. Moreover, dCAS appears to enable novice operators to achieve efficiency levels comparable to those of experienced clinicians, contributing to procedural standardization and efficiency irrespective of the operator’s experience [22]. However, other studies have not reported such improvements in the performance of novice operators using dCAS [23], raising doubts about how much this technology can support inexperienced clinicians. This variability suggests that improvements in efficiency and learning curves may depend more on the specific surgical procedure to which the technology is applied, as well as on individual operator’s adaptability, rather than on the technology itself.

Accuracy is among the most critical parameters for assessing the performance of dCAS. In coronectomies, Zhang et al. [18] reported a RMS deviation of 0.69 ± 0.21 mm, with approximately 72% of cases demonstrating deviations of less than 1 mm. These findings, although derived from a single pilot clinical study, suggest a high level of agreement between the virtual surgical plan and the actual outcome. From a clinical perspective, the magnitude of these deviations appears to fall within commonly accepted safety thresholds for guided oral surgical procedures. In dental implantology, maintaining a safety margin of approximately 1.5–2 mm [31,32] from the inferior alveolar nerve is generally considered critical to minimize the risk of neurosensory complications. Within the context of coronectomy procedure, the reported accuracy in this review suggests that dCAS may contribute to improved surgical predictability and a reduced likelihood of inadvertent nerve injury or the need for secondary surgical interventions.

This accuracy is consistent with the values reported for implant placement using dCAS, as described in studies by Block et al. [33] and Sun et al. [29], which demonstrated global deviations ranging from 0.5 to 1 mm and angular deviations below 4°. These results may be associated with the low risk of adverse effects observed when using dCAS. Such findings could help explain the emerging trend toward improved procedural safety with this technology, particularly when applied by experienced clinicians.

In endodontic surgery, the findings follow a similar trend in terms of accuracy and procedural safety. Aldahmash et al. [19] and Martinho et al. [20,23] reported lower 3D deviations and angular deflections in the groups treated with dCAS. For instance, in the study by Martinho et al. [20], angular deflection was reduced by more than 70%, from 9.4° in the FH group to 2.6° in the dCAS group. Likewise, global deviations at both the apex and the entry point were nearly halved compared to the FH technique.

In terms of clinical relevance, angular deviations exceeding 8–10° and millimetric inaccuracies during osteotomy or root-end resection may increase the risk of excessive bone removal, root perforation, or inadequate apical access, potentially compromising healing and long-term outcomes [34]. In this context, the accuracy levels below 3° and substantially reduced linear errors reported for dCAS seem to be within clinically acceptable values and may contribute to more conservative osteotomies and improved surgical predictability.

This trend is consistent across the remaining studies. An earlier investigation [23] also analyzed the influence of operator experience on procedural accuracy. The difference between EE and NE was smaller within the dCAS group than in the FH group, suggesting that the system may help reduce inter-operator variability and partially mitigate the impact of limited experience in complex procedures. Villa-Machado et al. [24] noted that a significant proportion of complications in endodontic surgery are due to imprecise manipulation during osteotomy or RER, often associated with insufficient operator experience. Taken together, these findings suggest that dCAS may function both as a performance-enhancing tool for experienced clinicians and as a safety-enhancing system for novice operators, although it does not replace the need for adequate training. In fact, one of the limitations of dCAS is the existence of a learning curve, which appears to vary depending on the type of procedure and the clinician’s level of experience. In implant placement, it has been suggested that operators may require between 10 and 20 procedures to achieve optimal accuracy [33]. A similar learning curve has been documented in endodontic surgery, where operators with no prior experience in dCAS were able to achieve consistent performance after 7 exercises [35]. Although dCAS can significantly improve the accuracy of NE operators compared to FH techniques, studies indicate that NE users do not reach the same performance levels as EE without an initial adaptation period [22,23,24]. Factors such as familiarity with the planning software, visuomotor coordination, and confidence in the technique are key contributors to this process. These findings highlight the need for proper training before using the system in clinical practice, to achieve good accuracy and effective procedures [24].

This systematic review has some limitations. Evidence on dCAS outside implantology is still scarce, and largely experimental. Moreover, most included studies present a moderate to high risk of bias, including selection bias, lack of blinding, and confounding in NRCTs, which reduces the certainty of the reported outcomes and should be considered when interpreting the observed effects on accuracy and efficiency. In addition, the included studies vary widely in design, surgical procedures, and the dCAS system employed, which makes direct comparison difficult. Future research should explore how operators learn specific procedures and how task load affects performance during interventions, to guide the development of clinical training programs.

## 5. Conclusions

Considering the limitations of this systematic review, the following conclusions can be drawn:

1. dCAS significantly improves the accuracy of endodontic procedures compared to conventional FH techniques under experimental (in vitro and cadaveric) conditions.
2. In endodontic surgery, dCAS contributes to reduced durations in critical phases such as osteotomy and RER in experimental models.
3. The complication rates observed in complex extractions and endodontic procedures using dCAS are comparable to those reported with FH techniques where clinical data exist.
4. The use of dCAS appears to reduce the influence of the operator’s experience on surgical accuracy, contributing to greater consistency across skill levels.
5. Current evidence is limited, heterogeneous, and mainly experimental. Clinical recommendations for routine oral surgical use of dCAS cannot yet be made outside selected indications. Further well-designed clinical studies are recommended to validate the applicability and reliability of dCAS in routine clinical practice.

## Figures and Tables

**Table 1:** PICO(S) question and inclusion criteria.

PICO(S)	Inclusion Criteria
Population (P)	Patients, in vitro experimental models, and cadaver studies.
Intervention (I)	Use of dCAS in oral surgical procedures, excluding implant or miniscrew placement.
Comparison (C)	Conventional FH techniques.
Outcomes (O)	Surgical accuracy, procedure time, and complication rate.
Study design (S)	RCTs, NRCTs with postoperative follow-up, cohort studies, case series (>10 patients), in vitro studies, and cadaver studies.

Abbreviations: RCTs: Randomized Controlled Trials; NRCTs: Non-Randomized Controlled Trials; dCAS: Dynamic Computer-Assisted Surgery; FH: Freehand.

**Table 2:** Search strategy and databases.

Database	Search Strategy (12 February to 18 March 2025)
PubMed (MEDLINE)	((“Dynamic Computer-Assisted Surgery” [Title/Abstract] OR “Dynamic Navigation” [Title/Abstract] OR “Real-Time Navigation” [Title/Abstract]) AND (“Oral Surgical Procedures” [Mesh Terms] OR “Dentoalveolar Surgery” [Title/Abstract] OR “Third Molar” [Title/Abstract] OR “Coronectomies” [Title/Abstract] OR “Extractions” [Title/Abstract])) NOT (“Dental Implants” [MesSH Terms] OR “Implant Surgery” [Title/Abstract])
Cochrane Library (Wiley)	(“Dynamic Computer-Assisted Surgery” OR “Dynamic Navigation” OR “Real-Time Navigation”) AND (“Oral Surgical Procedures” OR “Dentoalveolar Surgery” OR “Coronectomies” OR “Extractions” OR “Third Molar”) NOT (“Dental Implants” OR “Implant Surgery”)
Scopus (Elsevier)	TITLE-ABS-KEY (“Dynamic Computer-Assisted Surgery” OR “Dynamic Navigation” OR “Real-Time Navigation”) AND TITLE-ABS-KEY (“Oral Surgical Procedures” OR “Dentoalveolar Surgery” OR “Coronectomies” OR “Extractions” OR “Third Molar”) AND NOT TITLE-ABS-KEY (“Dental Implants” OR “Implant Surgery”)

**Table 3:** Characteristics of the included studies.

Study	Design	*n*	Sex (M/F)	Mean Age	Intervention	Evaluation
Wang et al. (2021) [15]	RCT	dCAS = 12 (No. of extractions = 16) FH = 12 (No. of extractions = 16)	15/1 14/2	8 (7–16) 9 (7–28)	Removal of impacted ST in the maxilla	Time (positioning and total), accuracy, complications
Emery et al. (2017) [16]	Retrospective review	dCAS = 21 (No. of extractions = 25)	14/7	47 (27–72)	Complex M3M extractions	IAN and LN injury, postoperative infection, trismus, mandibular fracture, major bleeding
Xu et al. (2024) [17]	RCT	dCAS = 80 FH = 80	5/45 39/41	23.7 (18–35) 24.54 (20–37)	Extraction of deeply impacted horizontal M3M	Time (preoperative planning, navigation setup, surgical), adjacent tooth damage, IAN and LN injury
Zhang et al. (2023) [18]	Non-randomized pilot study	dCAS = 12 (No. of extractions = 13)	5/7	28.67 (18–40)	M3M coronectomy	Coronectomy accuracy, lower lip paresthesia, infection, pulpitis symptoms, dry socket, root eruption
Aldahmash et al. (2022) [19]	Cadaver study (randomized, split-mouth design)	dCAS = 24, FH = 24	-	-	Osteotomy and RER	Accuracy (2D/3D deviation, angular deflection), osteotomy and resection metrics, RECP/REF viability, procedural time, incidents
Martinho et al. (2023) [20]	In vitro study (3D models, split-mouth design)	dCAS = 24, FH = 24	-	-	Osteotomy and RER	Accuracy (2D/3D deviation, angular deflection), RER accuracy, total operative time, incidents
Liu et al. (2024) [21]	In vitro study (3D models)	dCAS = 20, FH = 20 (40 replicas of an upper incisor)	-	-	RER in anterior teeth	Accuracy (length and angle deviation), efficiency, operative time
Tang and Jiang (2023) [22]	In vitro study (3D models)	dCAS-EE = 12/dCAS-NE = 12 sCAS-EE = 12/sCAS-NE = 12 FH-EE = 12/FH-NE = 12	-	-	RER (by EE and NE operators)	Accuracy (length, angle, volume, depth deviation), efficiency, safety, operative time, incidents, effect of operator experience
Martinho et al. (2022) [23]	Cadaver study (randomized)	dCAS-EE = 19/dCAS-NE = 19 FH-EE = 19/FH-NE = 19	-	-	Osteotomy and RER (by EE and NE operators)	Accuracy (deviation, angular deflection), operative time, incidents, operator experience
Villa-Machado et al. (2025) [24]	In vitro study	dCAS-EE = 60 dCAS-NE = 60	-	-	Osteotomy and RER (by EE and NE operators)	Accuracy (buccal entry point, apex, angle, depth), task performance, operator perception

Abbreviations: RCT: Randomized controlled trials; *n*: Sample size; M: Male; F: Female; dCAS: Dynamic computer-assisted surgery; FH: Freehand; ST: Supernumerary tooth; M3M: Mandibular third molar; IAN: Inferior alveolar nerve; LN: Lingual nerve; RER: Root-end resection; 3D: Three-dimensional; 2D: Two-dimensional; RECP: Root-end cavity preparation; REF: Root-end filling; sCAS: Static computer-assisted surgery; EE: Expert; NE: Novice.

**Table 4:** Efficiency in the included studies.

Study	Preoperative Design Time	Navigation Setup Time	Osteotomy + RER Time	Operative Time	Total Time
Tooth extraction procedures					
Xu et al. (2024) [17]	dCAS = 11 ± 1 min FH = 0 min	dCAS = 4 ± 1 min FH = 0 min	–	dCAS = 22 ± 3 min * FH = 36 ± 3 min *	dCAS = 37 ± 5 min FH = 36 ± 3 min
Zhang et al. (2023) [18]	ND	dCAS = 10–15 min	–	dCAS = 30–40 min	ND
Wang et al. (2021) [15]	ND	dCAS = 130 (40–663) s FH = 158 (10–1136) s	–	dCAS = 554.5 (210–1109) s FH = 498.5 (110–2079) s	ND
Endodontic surgery					
Aldahmash et al. (2022) [19]	ND	ND	dCAS = 550 ± 264 s * FH = 1167.5 ± 393 s *	dCAS = 800 ± 271 s * FH = 1423 ± 490 s *	ND
Martinho et al. (2023) [20]	ND	ND	dCAS = 220 ± 77 s * FH = 591 ± 60 s *	ND	ND
Tang and Jiang (2023) [22]	dCAS EE = 58.67 ± 2.57 s FH EE = 60.33 ± 3.14 s dCAS NE = 59.92 ± 2.91 s * FH NE = 69.33 ± 4.08 s *	ND	dCAS EE = 59.00 ± 4.10 s FH EE = 63.42 ± 4.78 s dCAS NE = 88.42 ± 8.63 s * FH NE = 120.75 ± 26.61 s *	dCAS EE = 117.67 ± 5.88 s FH EE = 123.75 ± 6.34 s dCAS NE = 148.33 ± 10.17 s * FH NE = 190.08 ± 26.62 s *	–
Liu et al. (2024) [21]	ND	ND	dCAS = 187 ± 22.97 s * FH = 247 ± 61.47 s *	ND	ND
Martinho et al. (2022) [23]	ND	ND	dCAS EE = 257 ± 90 s * FH EE = 540 ± 495 s * dCAS NE = 460 ± 270 s * FH NE = 1023 ± 408 s *	ND	ND

Abbreviations: RER: Root-end resection; dCAS: Dynamic computer-assisted surgery; FH: Freehand; –: Not applicable; min: Minutes; ND: No data available; s: Seconds; EE: Expert; NE: Novice; *: Significant difference between dCAS and FH groups (*p* < 0.05).

**Table 5:** Accuracy in endodontic surgery studies.

Study	2D Depth Deviation at Entry Point (mm)	2D Depth Deviation at Apex (mm)	3D Global Deviation at Entry Point (mm)	3D Global Deviation at Apex (mm)	Angular Deflection (°)
Aldahmash et al. (2022) [19]	dCAS = 1.09 ± 1.40 * FH = 1.56 ± 1.26 *	dCAS = 1.26 ± 1.39 * FH = 1.45 ± 1.28 *	dCAS = 0.60 ± 0.18 * FH = 1.29 ± 1.15 *	dCAS = 1.07 ± 1.55 * FH = 2.57 ± 1.68 *	dCAS = 1.10 ± 0.78 * FH = 16.03 ± 6.51 *
Martinho et al. (2023) [20]	dCAS = 0.80 ± 0.37 * FH = 1.41 ± 0.92 *	dCAS = 0.93 ± 0.55 * FH = 1.55 ± 1.07 *	dCAS = 0.72 ± 0.20 * FH = 1.29 ± 0.60 *	dCAS = 0.61 ± 0.30 * FH = 1.35 ± 0.82 *	dCAS = 2.60 ± 1.20 * FH = 9.40 ± 2.70 *
Tang and Jiang (2023) [22]	dCAS EE = 0.21 ± 0.18 * FH EE = 0.68 ± 0.31 * dCAS NE = 0.28 ± 0.09 * FH NE = 1.21 ± 0.61 *	dCAS EE = 0.45 ± 0.30 * FH EE = 1.36 ± 0.61 * dCAS NE = 0.53 ± 0.36 * FH NE = 1.91 ± 0.47 *	ND	ND	dCAS EE = 6.34 ± 4.81 * FH EE = 16.20 ± 6.67 * dCAS NE = 7.18 ± 4.43 * FH NE = 20.45 ± 5.48 *
Liu et al. (2024) [21]	dCAS = 0.46 ± 0.06 * FH = 1.20 ± 0.92 *	ND	ND	ND	dCAS = 2.45 ± 0.96 * FH = 16.20 ± 9.59 *
Martinho et al. (2022) [23]	dCAS EE = 0.80 ± 0.30 *ᵟ FH EE = 1.50 ± 1.30 * dCAS NE = 1.70 ± 0.60 *ᵟ FH NE = 2.10 ± 1.30 *	dCAS EE = 0.78 ± 0.50 *ᵟ FH EE = 1.30 ± 1.00 * dCAS NE = 1.50 ± 1.10 *ᵟ FH NE = 2.40 ± 1.00 *	dCAS EE = 0.70 ± 0.20 *ᵟ FH EE = 2.10 ± 0.30 * dCAS NE = 1.00 ± 0.40 *ᵟ FH NE = 5.70 ± 2.90 *	dCAS EE = 0.66 ± 0.50 *ᵟ FH EE = 2.70 ± 1.90 * dCAS NE = 1.20 ± 0.50 *ᵟ FH NE = 5.30 ± 1.50 *	dCAS EE = 1.30 ± 0.90 *ᵟ FH EE = 11.20 ± 2.40 * dCAS NE = 2.50 ± 0.80 *ᵟ FH NE = 15.40 ± 6.00 *
Villa-Machado et al. (2025) [24]	dCAS EE = 3.13 ± 2.50 *ᵟ dCAS NE = 5.86 ± 5.18 *ᵟ	dCAS EE = 3.04 ± 2.04 *ᵟ dCAS NE = 6.13 ± 5.01 *ᵟ	ND	ND	dCAS EE = 11.16 ± 7.87 *ᵟ dCAS NE = 16.77 ± 10.43 *ᵟ

Abbreviations: dCAS: Dynamic computer-assisted surgery; FH: Freehand; ND: No data available; EE: Expert; NE: Novice; *: Significant difference between dCAS and FH groups (*p* < 0.05); ᵟ: Significant difference between EE and NE groups (*p* < 0.05).

**Table 6:** Incidents and complications reported in tooth extraction and endodontic surgery studies (dCAS vs. FH).

Study	Procedure	Complications (*n*)	Reporting Data	Other Information
Xu et al. (2024) [17]	Extraction	dCAS = 0 FH = 7	Unclear	FH: Adjacent tooth damage (*n* = 3) and nerve injury (*n* = 4)
Emery et al. (2017) [16]	Extraction	dCAS = 12	Per-patient	dCAS: Nerve injury (*n* = 3), postoperative infection (*n* = 7, 2 pre-existing) and other complications (*n* = 2)
Zhang et al. (2023) [18]	Extraction	dCAS = 0	-	No complications reported
Aldahmash et al. (2022) [19]	Endodontic surgery	dCAS = 1 FH = 2	Per-procedure	dCAS: Incomplete apical resection (*n* = 1). FH: Sinus perforation (*n* = 1) and partially transected mental nerve (*n* = 1).
Martinho et al. (2023) [20]	Endodontic surgery	dCAS = 1 FH = 3	Unclear	Data not reported
Tang and Jiang (2023) [22]	Endodontic surgery	dCAS EE = 0; FH EE = 1 dCAS NE = 0; FH NE = 5	Per-procedure	FH EE: Maxillary sinus perforation (*n* = 1). FH NE: Maxillary sinus perforation (*n* = 4) and incomplete apical resection (*n* = 1)
Martinho et al. (2022) [23]	Endodontic surgery	dCAS EE = 1; FH EE = 2 dCAS NE = 1; FH NE = 2	Per-procedure	Data not reported
Villa-Machado et al. (2025) [24]	Endodontic surgery	dCAS EE = 18 ᵟ dCAS NE = 32 ᵟ	Per-procedure	Data not reported

Abbreviations: dCAS: Dynamic computer-assisted surgery; FH: Freehand, EE: Expert; NE: Novice; ᵟ: Significant difference between EE and NE groups (*p* < 0.05).

## Data Availability

Data are contained within the article.

## Supplementary Materials

The following supporting information can be downloaded at: https://www.mdpi.com/article/10.3390/jcm15020886/s1, Table S1: Equipment, registration methods, and software used in the included studies; Table S2: Description of risk of bias in randomized controlled trials (RCTs) according to the RoB2 Cochrane Collaboration tool (version 5.0.1, 2011); Table S3: Description of risk of bias according to the ROBINS-I tool for non-randomized studies (NRS); Table S4: PRISMA 2020 Abstract Checklist; Table S5: PRISMA 2020 Checklist.

## Abbreviations

The following abbreviations are used in this manuscript:

CBCT	Cone beam computed tomography
CAS	Computer-assisted surgery
3D	Three-dimensional
2D	Two-dimensional
sCAS	Static computer-assisted surgery
dCAS	Dynamic computer-assisted surgery
FH	Freehand
PRISMA	Preferred Reporting Items for Systematic Reviews and Meta-Analyses
RCTs	Randomized controlled trials
NRCTs	Non-randomized controlled trials
*n*	Sample size
M3M	Mandibular third molar
IAN	Inferior alveolar nerve
LN	Lingual nerve
ST	Supernumerary tooth
RER	Root-end resection
RECP	Root-end cavity preparation
REF	Root-end filling
EE	Expert
NE	Novice
IGI	Image-guided implantology
NRS	Non-randomized studies
RMS	Root mean square
