# Supplementary material for: Dynamic Computer-Assisted Surgery in Oral Surgery: A Systematic Review
Source: J Clin Med. 2026 Jan 21;15(2):886. doi: 10.3390/jcm15020886 (PMC12842480; doi:10.3390/jcm15020886)
Supplement: Supplementary file 1 [file jcm-15-00886-s001.zip › jcm-4074078-supplementary.pdf]

**Table S1.** Equipment, registration methods, and software used in the included studies.

| Study | Imaging System | Surgical Instruments | Navigation System | Registration Method | Software |
|---|---|---|---|---|---|
| Wang et al. (2021) [15] | CBCT | High-speed turbine | Brainlab ENT/CMF system | Modified occlusal registration device | iPlan CMF 2.1 (Brainlab, Germany) |
| Emery et al. (2017) [16] | CBCT | Custom straight turbine with integrated tracking array | IGI system (DenX Advanced Dental Systems, Israel) | Custom-fabricated splint with fiducial reference frame | Navigation software (DenX Advanced Dental Systems) |
| Xu et al. (2024) [17] | CBCT | High-speed turbine (NSK, Japan) | DHC-DI2 system (Dekaier, China) | Right M3M: 13 M + C24; Left M3M: 24 M + C13 (Dekaier, China) | MIMICS 21.0 (Belgium) |
| Zhang et al. (2023) [18] | CBCT | High-speed turbine with long and short spherical burs | Dexter® system | U-shaped registration device fixed to mandibular dentition | Mimics™ 19.0 (Materialise Dental, Belgium); GeoMagic™ Studio 12 (3D Systems, USA) |
| Aldahmash et al. (2022) [19] | CBCT | 10 mm precision trephine + 3.5 mm bone drill (Nobel Biocare, USA) | X-Guide® system | Clip with 3 radiopaque markers (X-Nav Technologies, USA), placed on molar dentition | X-Guide® (X-Nav Technologies, USA); Mimics Innovation Suite (Materialise NV, Belgium) |
| Martinho et al. (2023) [20] | CBCT | 3.5 mm Ø surgical drill (Nobel Biocare, USA) | X-Guide® system | X-clip with radiopaque markers (X-Nav Technologies), adapted to contralateral molars | X-Guide® (X-Nav Technologies, USA); virtual surgical planning with CBCT overlay |
| Liu et al. (2024) [21] | CBCT | 4.5 mm Ø surgical trephines | DCARER system (Suzhou, China) | Silicone registration device | DCARER |
| Tang and Jiang (2023) [22] | CBCT | 4 mm Ø trephine | DCARER system (Suzhou, China) | Tracking device fixed to non-operative dentition and handpiece | DCARER; Mimics v20.0 and 3-matic Medical v12.0 (Materialise, Belgium) |
| Martinho et al. (2022) [23] | CBCT | 3.5 mm Ø surgical drill (Nobel Biocare, USA) | X-Guide® system (ClaroNav, Canada) | X-clip with radiopaque markers placed on contralateral molars | X-Guide® planning software; X-Nav Technologies overlay software (USA) |
| Villa-Machado et al. (2025) [24] | CBCT | 4–5 mm Ø surgical trephines | Navident® system (ClaroNav, Canada) | Jaw-Tracker device fixed with dual-cure resin cement | Navident® and EvaluNav® planning software (ClaroNav) |

Abbreviations: CBCT: Cone beam computed tomography; IGI: Image-guided implantology; dCAS: Dynamic computer-assisted surgery; M3M: Mandibular third molar.

**Table S2.** Description of risk of bias in randomized controlled trials (RCTs) according to the RoB2 Cochrane Collaboration tool (version 5.0.1, 2011).

| Authors | Selection Bias | | Performance Bias | Detection Bias | Attrition Bias | Reporting Bias |
|---|---|---|---|---|---|---|
| | Generation of the Random Sequence | Allocation Concealment | Blinding of Personnel and Participants | Blinding of Outcome Assessors | Incomplete Outcome Data | Selective Reporting of Outcomes |
| Wang et al. (2021) [15] | + | + | - | + | + | + |
| Xu et al. (2024) [17] | + | ! | - | ! | + | + |
| Aldahmash et al. (2022) [19] | + | ! | - | + | + | + |
| Martinho et al. (2022) [23] | + | + | - | + | + | + |

Abbreviations: Red: high risk of bias, yellow: unclear risk of bias, green: low risk of bias.

**Table S3.** Description of risk of bias according to the ROBINS-I tool for non-randomized studies (NRS).

| Authors | Bias due to Confounding | Bias in Selection of Participants | Bias in Classification of Interventions | Bias due to Deviations from Intended Interventions | Bias due to Missing Data | Bias in Measurement of Outcomes | Bias in Selection of Reported Results | Overall Risk of Bias |
|---|---|---|---|---|---|---|---|---|
| Emery et al. (2017) [16] | Serious | Serious | Low | Low | Low | Moderate | Moderate | Serious |
| Zhang et al. (2023) [18] | Serious | Moderate | Low | Low | Moderate | Moderate | Serious | Serious |

**Table S4.** PRISMA 2020 for Abstracts Checklist.

| Section and Topic | Item # | Checklist item | Reported (Yes/No) |
|---|---|---|---|
| **TITLE** | | | |
| Title | 1 | Identify the report as a systematic review. | yes |
| **BACKGROUND** | | | |
| Objectives | 2 | Provide an explicit statement of the main objective(s) or question(s) the review addresses. | yes |
| **METHODS** | | | |
| Eligibility criteria | 3 | Specify the inclusion and exclusion criteria for the review. | yes |
| Information sources | 4 | Specify the information sources (e.g. databases, registers) used to identify studies and the date when each was last searched. | yes |
| Risk of bias | 5 | Specify the methods used to assess risk of bias in the included studies. | yes |
| Synthesis of results | 6 | Specify the methods used to present and synthesise results. | yes |
| **RESULTS** | | | |
| Included studies | 7 | Give the total number of included studies and participants and summarise relevant characteristics of studies. | yes |
| Synthesis of results | 8 | Present results for main outcomes, preferably indicating the number of included studies and participants for each. If meta-analysis was done, report the summary estimate and confidence/credible interval. If comparing groups, indicate the direction of the effect (i.e. which group is favoured). | yes |
| **DISCUSSION** | | | |
| Limitations of evidence | 9 | Provide a brief summary of the limitations of the evidence included in the review (e.g. study risk of bias, inconsistency and imprecision). | yes |
| Interpretation | 10 | Provide a general interpretation of the results and important implications. | yes |
| **OTHER** | | | |
| Funding | 11 | Specify the primary source of funding for the review. | no |
| Registration | 12 | Provide the register name and registration number. | no |

**Table S5.** PRISMA 2020 Checklist.

| Section and Topic | Item # | Checklist item | Location where item is reported |
|---|---|---|---|
| **TITLE** | | | |
| Title | 1 | Identify the report as a systematic review. | Page 1 |
| **ABSTRACT** | | | |
| Abstract | 2 | See the PRISMA 2020 for Abstracts checklist. | Page 1-2 |
| **INTRODUCTION** | | | |
| Rationale | 3 | Describe the rationale for the review in the context of existing knowledge. | Page 2-3 |
| Objectives | 4 | Provide an explicit statement of the objective(s) or question(s) the review addresses. | Page 3 |
| **METHODS** | | | |
| Eligibility criteria | 5 | Specify the inclusion and exclusion criteria for the review and how studies were grouped for the syntheses. | Page 3 |
| Information sources | 6 | Specify all databases, registers, websites, organisations, reference lists and other sources searched or consulted to identify studies. Specify the date when each source was last searched or consulted. | Page 4 |
| Search strategy | 7 | Present the full search strategies for all databases, registers and websites, including any filters and limits used. | Page 4 |
| Selection process | 8 | Specify the methods used to decide whether a study met the inclusion criteria of the review, including how many reviewers screened each record and each report retrieved, whether they worked independently, and if applicable, details of automation tools used in the process. | Page 4 |
| Data collection process | 9 | Specify the methods used to collect data from reports, including how many reviewers collected data from each report, whether they worked independently, any processes for obtaining or confirming data from study investigators, and if applicable, details of automation tools used in the process. | Page 4 |
| Data items | 10a | List and define all outcomes for which data were sought. Specify whether all results that were compatible with each outcome domain in each study were sought (e.g. for all measures, time points, analyses), and if not, the methods used to decide which results to collect. | Page 4 |
| | 10b | List and define all other variables for which data were sought (e.g. participant and intervention characteristics, funding sources). Describe any assumptions made about any missing or unclear information. | Page 4 |
| Study risk of bias assessment | 11 | Specify the methods used to assess risk of bias in the included studies, including details of the tool(s) used, how many reviewers assessed each study and whether they worked independently, and if applicable, details of automation tools used in the process. | Page 4 |
| Effect measures | 12 | Specify for each outcome the effect measure(s) (e.g. risk ratio, mean difference) used in the synthesis or presentation of results. | Not applicable (no meta-analysis performed). |

| Section and Topic | Item # | Checklist item | Location where item is reported |
|---|---|---|---|
| | | | |
| Synthesis methods | 13a | Describe the processes used to decide which studies were eligible for each synthesis (e.g. tabulating the study intervention characteristics and comparing against the planned groups for each synthesis (item #5)). | Page 4–5 |
| | 13b | Describe any methods required to prepare the data for presentation or synthesis, such as handling of missing summary statistics, or data conversions. | Page 4 |
| | 13c | Describe any methods used to tabulate or visually display results of individual studies and syntheses. | Page 4 |
| | 13d | Describe any methods used to synthesize results and provide a rationale for the choice(s). If meta-analysis was performed, describe the model(s), method(s) to identify the presence and extent of statistical heterogeneity, and software package(s) used. | Page 4 |
| | 13e | Describe any methods used to explore possible causes of heterogeneity among study results (e.g. subgroup analysis, meta-regression). | Not applicable (no meta-analysis performed) |
| | 13f | Describe any sensitivity analyses conducted to assess robustness of the synthesized results. | Not applicable (no meta-analysis performed) |
| Reporting bias assessment | 14 | Describe any methods used to assess risk of bias due to missing results in a synthesis (arising from reporting biases). | Not applicable (no statistical synthesis conducted) |
| Certainty assessment | 15 | Describe any methods used to assess certainty (or confidence) in the body of evidence for an outcome. | Not applicable (GRADE not |

| Section and Topic | Item # | Checklist item | Location where item is reported |
|---|---|---|---|
| | | | performed due to absence of meta-analysis) |
| **RESULTS** | | | |
| Study selection | 16a | Describe the results of the search and selection process, from the number of records identified in the search to the number of studies included in the review, ideally using a flow diagram. | Page 5 + PRISMA flow diagram |
| | 16b | Cite studies that might appear to meet the inclusion criteria, but which were excluded, and explain why they were excluded. | Page 5 |
| Study characteristics | 17 | Cite each included study and present its characteristics. | Page 6 |
| Risk of bias in studies | 18 | Present assessments of risk of bias for each included study. | Page 7 |
| Results of individual studies | 19 | For all outcomes, present, for each study: (a) summary statistics for each group (where appropriate) and (b) an effect estimates and its precision (e.g. confidence/credible interval), ideally using structured tables or plots. | Page 6-10 |
| Results of syntheses | 20a | For each synthesis, briefly summarise the characteristics and risk of bias among contributing studies. | Page 7-12 |
| | 20b | Present results of all statistical syntheses conducted. If meta-analysis was done, present for each the summary estimate and its precision (e.g. confidence/credible interval) and measures of statistical heterogeneity. If comparing groups, describe the direction of the effect. | Page 8-11 |
| | 20c | Present results of all investigations of possible causes of heterogeneity among study results. | Page 7-12 |
| | 20d | Present results of all sensitivity analyses conducted to assess the robustness of the synthesized results. | Not applicable |
| Reporting biases | 21 | Present assessments of risk of bias due to missing results (arising from reporting biases) for each synthesis assessed. | Not applicable |
| Certainty of evidence | 22 | Present assessments of certainty (or confidence) in the body of evidence for each outcome assessed. | Not applicable |
| **DISCUSSION** | | | |
| Discussion | 23a | Provide a general interpretation of the results in the context of other evidence. | Page 13 |
| | 23b | Discuss any limitations of the evidence included in the review. | Page 14 |
| | 23c | Discuss any limitations of the review processes used. | Page 14 |

| Section and Topic | Item # | Checklist item | Location where item is reported |
|---|---|---|---|
| | 23d | Discuss implications of the results for practice, policy, and future research. | Page 14 |
| **OTHER INFORMATION** | | | |
| Registration and protocol | 24a | Provide registration information for the review, including register name and registration number, or state that the review was not registered. | Page 3 |
| | 24b | Indicate where the review protocol can be accessed, or state that a protocol was not prepared. | Page 3 |
| | 24c | Describe and explain any amendments to information provided at registration or in the protocol. | Not applicable |
| Support | 25 | Describe sources of financial or non-financial support for the review, and the role of the funders or sponsors in the review. | Page 15 |
| Competing interests | 26 | Declare any competing interests of review authors. | Page 15 |
| Availability of data, code and other materials | 27 | Report which of the following are publicly available and where they can be found: template data collection forms; data extracted from included studies; data used for all analyses; analytic code; any other materials used in the review. | Page 15 |
